# Potential Role of Lysine Acetylation in Antibiotic Resistance of Escherichia coli

**DOI:** 10.1128/msystems.00649-22

**Published:** 2022-10-26

**Authors:** Zuye Fang, Fubin Lai, Kun Cao, Ziyuan Zhang, Linlin Cao, Shiqin Liu, Yufeng Duan, Xingfeng Yin, Ruiguang Ge, Qing-Yu He, Xuesong Sun

**Affiliations:** a MOE Key Laboratory of Tumor Molecular Biology, Institute of Life and Health Engineering, College of Life Science and Technology, Jinan University, Guangzhou, China; b Guangdong Provincial Key Laboratory of Medical Molecular Diagnostics, Institute of Aging Research, Guangdong Medical University, Dongguan, China; c Key Laboratory of Gene Engineering of the Ministry of Education, College of Life Sciences, Sun Yat-Sen University, Guangzhou, China; d Key Laboratory of Functional Protein Research of Guangdong Higher Education Institutes, Institute of Life and Health Engineering, College of Life Science and Technology, Jinan University, Guangzhou, China; e State Key Laboratory of Biocontrol, College of Life Sciences, Sun Yat-Sen University, Guangzhou, China; Leiden University

**Keywords:** post-translational modification, *Escherichia coli*, acetylation, PykF, antibiotic resistance

## Abstract

Antibiotic resistance is increasingly becoming a challenge to public health. The regulation of bacterial metabolism by post-translational modifications (PTMs) has been widely studied. However, the mechanism underlying the regulation of acetylation in bacterial resistance to antibiotics is still unknown. Here, we performed a quantitative analysis of the acetylated proteome of a wild-type (WT) Escherichia coli (E. coli) sensitive strain and ampicillin- (Re-Amp), kanamycin- (Re-Kan), and polymyxin B-resistant (Re-Pol) strains. Based on bioinformatics analysis combined with biochemical validations, we found a common regulatory mechanism between the different resistant strains. Our results showed that protein acetylation negatively regulates bacterial metabolism to regulate antibiotic resistance and positively regulates bacterial motility. Further analyses revealed that key enzymes in various metabolic pathways were differentially acetylated. In particular, pyruvate kinase (PykF), a glycolytic enzyme that regulates bacterial metabolism, and its acetylated form were highly expressed in the three resistant strains and were identified as reversibly acetylated by the deacetylase CobB and the acetyl-transferase PatZ (peptidyl-lysine *N*-acetyltransferase). Results showed that PykF also could be acetylated by nonenzymatic acetyl phosphatase (AcP) *in vitro*. Furthermore, the deacetylation of Lys413 in PykF increased PykF enzymatic activity by changing the conformation of its ATP binding site, resulting in an increase in energy production which, in turn, increased the sensitivity of drug-resistant strains to antibiotics. This study provides novel insights for understanding bacterial resistance and lays the foundation for future research on the regulation of acetylation in antibiotic-resistant strains.

**IMPORTANCE** The misuse of antibiotics has resulted in the emergence of many antibiotic-resistant strains which seriously threaten human health. Protein post-translational modifications, especially acetylation, tightly control bacterial metabolism. However, the comprehensive mechanism underlying the regulation of acetylation in bacterial resistance remains unexplored. Here, acetylation was found to positively regulate bacterial motility and negatively regulate energy metabolism, which was common in all antibiotic-resistant strains. Moreover, the acetylation and deacetylation process of PykF was uncovered, and deacetylation of the Lys 413 in PykF was found to contribute to bacterial sensitivity to antibiotics. This study provides a new direction for research on the development of bacterial resistance through post-translational modifications and a theoretical basis for developing antibacterial drugs.

## INTRODUCTION

Antibiotics are widely used to treat bacterial infections, improving quality of life and reducing mortality. Unfortunately, the frequent abuse of antibiotics has led to the rapid emergence of antibiotic-resistant strains which seriously threaten human health (1). Bacteria have developed different mechanisms to resist different antibiotics; for example, highly active penicillin-binding protein production to resist ampicillin (2), structural change in the 30S subunit to resist kanamycin (3, 4), and structural alteration of lipid A to combat polymyxin B (5, 6).

Protein post-translational modifications (PTMs) regulate many important biological pathways in bacteria. In recent years, with the development of mass spectrometry (MS) and enrichment strategies, numerous PTMs have been found to be related to bacterial metabolism (7–12). With ongoing research, many key kinases have been found to be regulated by acetylation. The extent of acetylation in E. coli varies at different growth stages, and it is higher in the stationary phase than in the logarithmic growth phase, suggesting that acetylation changes affect the bacterial metabolic state (13). Lysine acetylation is one of the most widely known forms of PTMs of proteins in bacteria and is involved in the regulation of bacterial transcription, translation, metabolism, stress response, and other important physiological processes (10, 14, 15). In E. coli, two mechanisms of Nε-lysine acetylation are known. The main mechanism is acetyl phosphate (AcP)-dependent nonenzymatic acetylation. AcP is an intermediate product of the phosphotransferase (Pta)-acetate kinase (AckA) pathway which can mutually convert acetyl coenzyme A (acetyl-CoA), inorganic phosphate, and ADP into acetate, CoA, and ATP. The secondary mechanism is an enzymatic reaction mediated by Nε-lysine acetyltransferase (KAT) and deacetylases (KDACs) (16). The most common KAT in bacteria is PatZ, also called Pka or YfiQ (16–18). In addition, four new types of KATs, RimI, YiaC, YjaB, and PhnO, were also discovered (19). NAD^+^-dependent CobB is considered the common KDAC in bacteria. When *cobB* of E. coli was deleted, acetylation levels were increased, and resistance ability to heat shock and oxidative stress was improved (15, 16). The same phenomenon has also been observed in mycobacteria (20).

In bacteria, the activity of multiple transcription factors, including RcsB, Lrp, and PhoP, can be regulated by acetylation (14, 21). Various acetylated proteins are also involved in bacteria’s important carbon metabolism pathways, such as the tricarboxylic acid (TCA) cycle, glycolysis, and oxidative phosphorylation (10, 15, 22). Thus, acetylation significantly influences the regulation of bacterial metabolism. In addition, acetylation also plays an important role in regulating bacterial pathogenicity and virulence (14); for example, in Salmonella spp., PhoP can be acetylated by Pat at lysine 201 (K201), and the deacetylation of PhoP K201 can ensure that the bacteria adapt to the oxidative stress derived from the host cells. One previous study indicates that the acetylation at K201 in PhoP is necessary for the pathogenicity of Salmonella spp.(21). Although regulation of bacterial metabolism by PTMs has been reported before, how lysine acetylation regulates bacterial resistance to antibiotics has not yet been deciphered.

This study aims to reveal a common mechanism for the regulation of bacterial antibiotic resistance to different types of antibiotics. Here, we used quantitative proteomics analysis combined with antibody enrichment to investigate how acetylation affects bacterial resistance to β-lactams (ampicillin), aminoglycosides (kanamycin), and polypeptides (polymyxin B). Furthermore, biochemical analyses were used to unravel the key biological functions of the specific sites acetylated in proteins to reveal novel comprehensive mechanisms of PTMs in regulating bacterial antibiotic resistance. This study could pave the way for developing new drugs to treat infections caused by drug-resistant strains.

## RESULTS

### Acetylation intensities in different antibiotic-resistant *Escherichia coli*.

**FIG 1:**
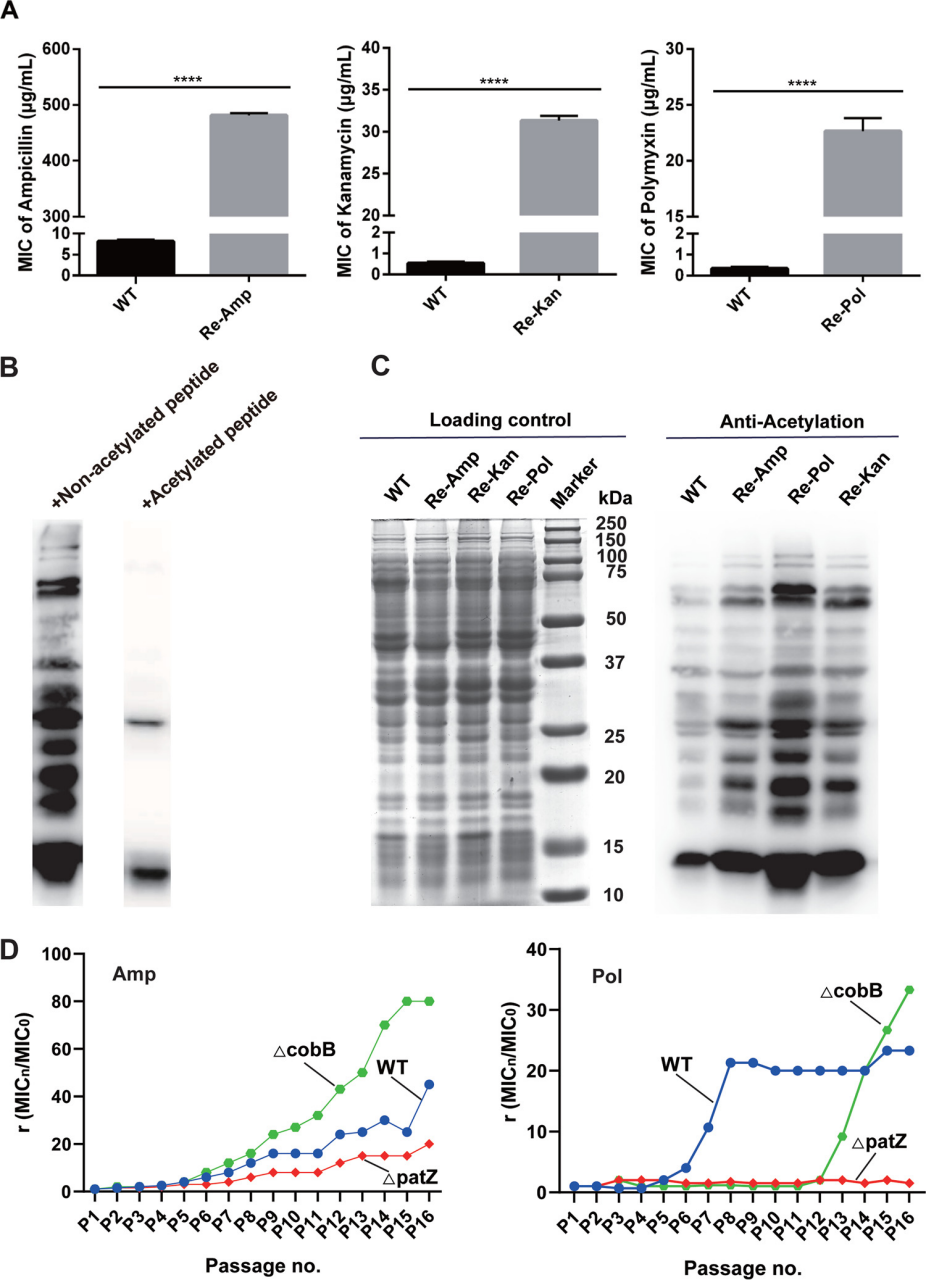
Relationship between acetylation modification and antibiotic resistance in Escherichia coli. (A) Average minimum inhibitory concentrations (MIC) of the highly resistant strains from left to right are shown. Ampicillin (Amp): wild-type (WT) strain = 8.2 μg/mL, Amp-resistant strain (Re-Amp) = 481.7 μg/mL. Kanamycin (Kan): WT = 0.5 μg/mL, Kan-resistant (Re-Kan) = 31.3 μg/mL. Polymyxin B (Pol): WT = 0.3 μg/mL, Pol-resistant (Re-Pol) = 22.6 μg/mL. Experiments were performed in triplicate. Two-tailed unpaired *t* test was used for statistical analysis. (*, *P* < 0.05; **, *P* < 0.01; ***, *P* < 0.005; ****, *P* < 0.001). (B) Acetylated antibodies were treated with acetylated or unmodified peptide libraries to detect their specificity. (C) Immunoblot of the Escherichia coli lysate with the α-AcK monoclonal antibody. (D) Resistance formation ability of Escherichia coli BW25113 and Δ*patZ* and Δ*cobB* strains to ampicillin (left) and polymyxin (right).

The differences in the acetylation intensities of the whole proteome between wild-type (WT) and antibiotic-resistant Escherichia coli were investigated. The E. coli BW25113 strain was continuously subcultured in media with ampicillin, kanamycin, or polymyxin B to generate strains highly resistant to the antibiotic classes of β-lactams, aminoglycosides, and polypeptides, respectively. Measurement of the minimum inhibitory concentration (MIC) showed that the MIC of the highly resistant bacterial strains was increased approximately 60-fold compared with that of the WT. For ampicillin, the MICs of WT and Re-Amp were 8.2 and 481.7 μg/mL, respectively; for kanamycin, the MICs of WT and Re-Kan were 0.5 and 31.3 μg/mL, respectively; and for polymyxin B, the MICs of WT and Re-Pol were 0.3 and 22.6 μg/mL, respectively (Fig. 1A).

We collected WT and the three antibiotic-resistant strains in the logarithmic growth period (optical density at 600 nm (OD_600_) of ~0.8) because bacterial metabolism was most vigorous during this period. Immunoblot with α-AcK monoclonal antibody, which has a good specificity validated by a competition experiment (Fig. 1B), indicated a difference in the acetylation intensities of the whole protein level between the WT and the three antibiotic-resistant strains (Fig. 1C). In particular, the proteins of the drug-resistant bacteria showed higher levels of acetylation than those of the WT, suggesting that protein acetylation may be conducive to the regulation of drug resistance. In addition, distinct drug-resistant strains showed specific profiles of acetylated proteins, indicating that bacteria resistant to different drugs may also have drug-specific resistance regulatory mechanisms. To further explore the role of acetylation modification in antibiotic resistance, we investigated the resistance development capacities of Δ*patZ* and Δ*cobB* to ampicillin and polymyxin B (Fig. 1D). Two knockout strains (National Institute of Genetics, Japan) have kanamycin-resistance genes acquired during the process of gene knockout; thus, we did not detect their kanamycin resistance. The results showed that the Δ*cobB* strain was more likely than the WT strain to develop antibiotic resistance. In contrast, the Δ*patZ* strain did not develop drug resistance easily, which may be due to the low acetylation levels of various proteins in the bacteria caused by the deletion of this important acetyltransferase. Interestingly, the resistance of the Δ*cobB* strain to polymyxin B was latent and increased suddenly, which may be related to the complicated mechanism of action of polymyxin B (23). This result indicates that acetylation may positively correlate with antibiotic resistance in bacteria.

### Distribution of acetylation in *Escherichia coli* is stable.

**FIG 2:**
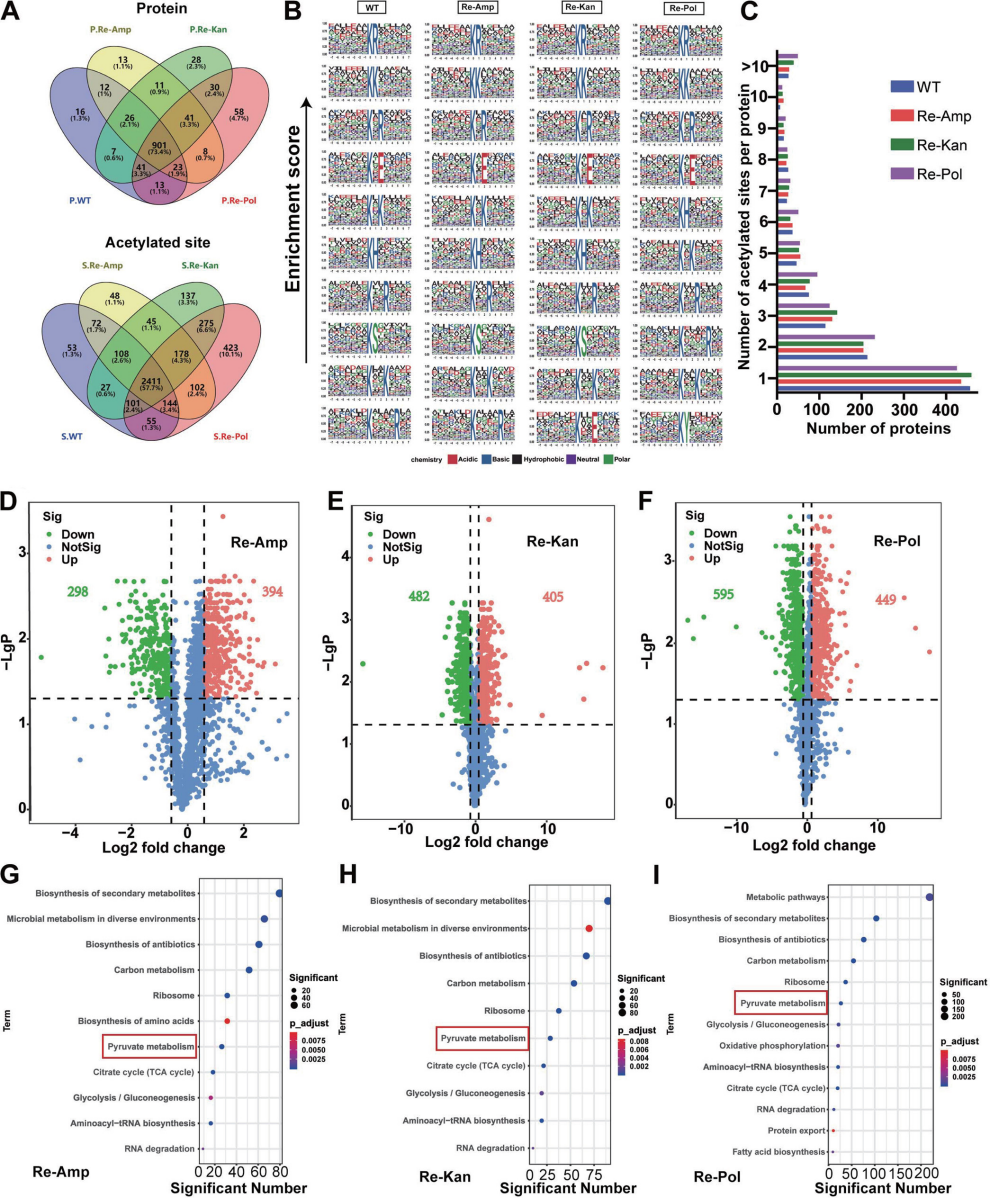
Acetylated proteins and sites in E. coli. (A) Statistics of acetylated proteins and acetylated sites in the different resistant types. P, acetylated protein; S, acetylated site. (B) Motif significant enrichment analysis on the identified acetylated peptides (7 upstream and downstream amino acids of acetylated lysine). From left to right: WT, Re-Amp, Re-Kan, and Re-Pol. *P* < 0.000001 was considered significant. (C) The number of acetylated sites identified per protein. (D to F) Volcano graphs for the statistical analysis of acetylation peptides (difference multiple is 1.5× up and down). (G to I) KEGG pathway analysis of the differentially acetylated proteins. *P* < 0.05. For panels A to I, *n* = 3 independent biological replicates.

To further study the relationship between acetylation and antibiotic resistance, we enriched acetylated peptides of the WT and the three antibiotic-resistant strains using α-AcK antibody-conjugated beads and then identified them by data-independent acquisition (DIA)-based liquid chromatography-tandem mass spectrometry (LC-MS/MS) analysis. The total distribution of mass error for precursor ions was <0.03 Da, indicating acceptable mass accuracy of the MS data. We identified 2,971 lysine-acetylated sites of 1,039 proteins in WT strain; 3,108 acetylated sites of 1,035 proteins in the Re-Amp strain; 3,282 acetylated sites of 1,085 proteins in the Re-Kan strain; and 3,689 lysine acetylated sites of 1,115 proteins in the Re-Pol strain. Hierarchical cluster analysis showed that the proteomic data had good biological reproducibility (*n* = 3, Fig. S1A and B). Our study showed higher enrichment efficiency and MS sensitivity than a previous acetyl-proteomic study in the same species, E. coli BW25113, which identified 2,803 acetylated sites of 809 proteins (24). We identified a total of 4,193 acetylated sites of 1,228 proteins in all samples, of which 2,411 (57.5%) sites of 901 (73.4%) proteins were commonly identified in the WT and all antibiotic-resistant strains, showing the stability of acetylation in bacteria (Fig. 2A). We further assessed the distribution of lysine-acetylated sites and found that most of the proteins were monoacetylated, which was in accordance with acetylome studies in other bacterial species (25–27). In addition, the distribution of monoacetylated and polyacetylated proteins was almost the same in the WT and resistant strains (Fig. 2C). The stable distribution of acetylation in E. coli in different resistant phenotypes exhibited the high conservation of this modification.

10.1128/msystems.00649-22.4 FIG S1 The reproducibility and representative mass spectra of proteomics data. (A) Hierarchical cluster analysis of proteins at the whole-cell level. (B) Heatmap analysis of quantitative data on acetylated peptides. (C to E) Heatmap of hierarchical cluster analysis for differential acetylated peptides. (F) Tandem mass spectrometry (MS/MS) spectra of three peptides, including experiments performed in triplicate. Download FIG S1, EPS file, 2.8 MB. Copyright © 2022 Fang et al. 2022 Fang et al. https://creativecommons.org/licenses/by/4.0/ This content is distributed under the terms of the Creative Commons Attribution 4.0 International license.

To determine the preference for lysine acetylation in different antibiotic-resistant strains, we used the Motif-X tool to perform sequence analysis of the acetylated peptides (seven upstream and seven downstream amino acids of the acetylated lysine). Eight motif sequences were overrepresented in all the identified peptides (Fig. 2B), including KR/K/H, KxK/R/E, and KxxK/R (K, the acetylated lysine; x, random amino acid residues). The amino acids in these motifs were hydrophilic, indicating that KATs prefer a hydrophilic environment and that the occurrence of acetylation sites is stable (18). Arginine, lysine, and histidine are preferentially located at the +1 position; lysine, arginine, and glutamic acid at the +2 position; and lysine and arginine at the +3 position. The +1 position is preferentially a positively charged amino acid, possibly because most acetylated proteins had AcP-dependent acetylation. This preference has also been found in other organisms; for example, the KH motif has been identified in humans, plants, and Vibrio parahaemolyticus (28–30), implying that this protein modification may be relatively conservative. Interestingly, new motifs were detected in this study: KxxE in Re-Kan and KY in Re-Pol, suggesting that bacteria have new substrate-specific KATs involved in the regulation of resistance due to the continuous pressure of kanamycin and polymyxin B.

### Drug resistance to different antibiotics may be regulated by acetylation.

To determine whether the changes in protein acetylation were related to drug resistance, the quantitative proteome data were used as the background. Statistical analysis of the different acetylated peptides (up and down 1.5-fold) in the three resistant strains showed that compared with the WT, Re-Amp had 366 differentially acetylated proteins (DAPs), including 692 differentially acetylated peptides. In contrast, Re-Kan had 455 DAPs, including 887 differentially acetylated peptides, and Re-Pol had 522 DAPs, including differentially 1,044 acetylated peptides (Fig. 2D to F). In addition, the heatmap results of the proteome study showed differential patterns of acetylated peptides between sensitive and drug-resistant bacteria (Fig. S1C to E); Re-Pol possessed more differentially expressed acetylated peptides.

Next, we conducted KEGG pathway enrichment analyses of the DAPs of the three resistant strains and found that the DAPs were significantly enriched in the ribosome, aminoacyl-tRNA biosynthesis, RNA degradation, pyruvate metabolism, methane metabolism, glycolysis, the TCA cycle, and oxidative phosphorylation (Fig. 2G to I). These findings are consistent with acetylation mainly occurring in the translation and metabolism of prokaryotes and eukaryotes (10). These results further indicate that regulation of bacterial resistance may be related to protein synthesis and energy metabolism regulated by acetylation.

### Acetylation reduces the metabolic activity of *E. coli* to regulate antibiotic resistance.

**FIG 3:**
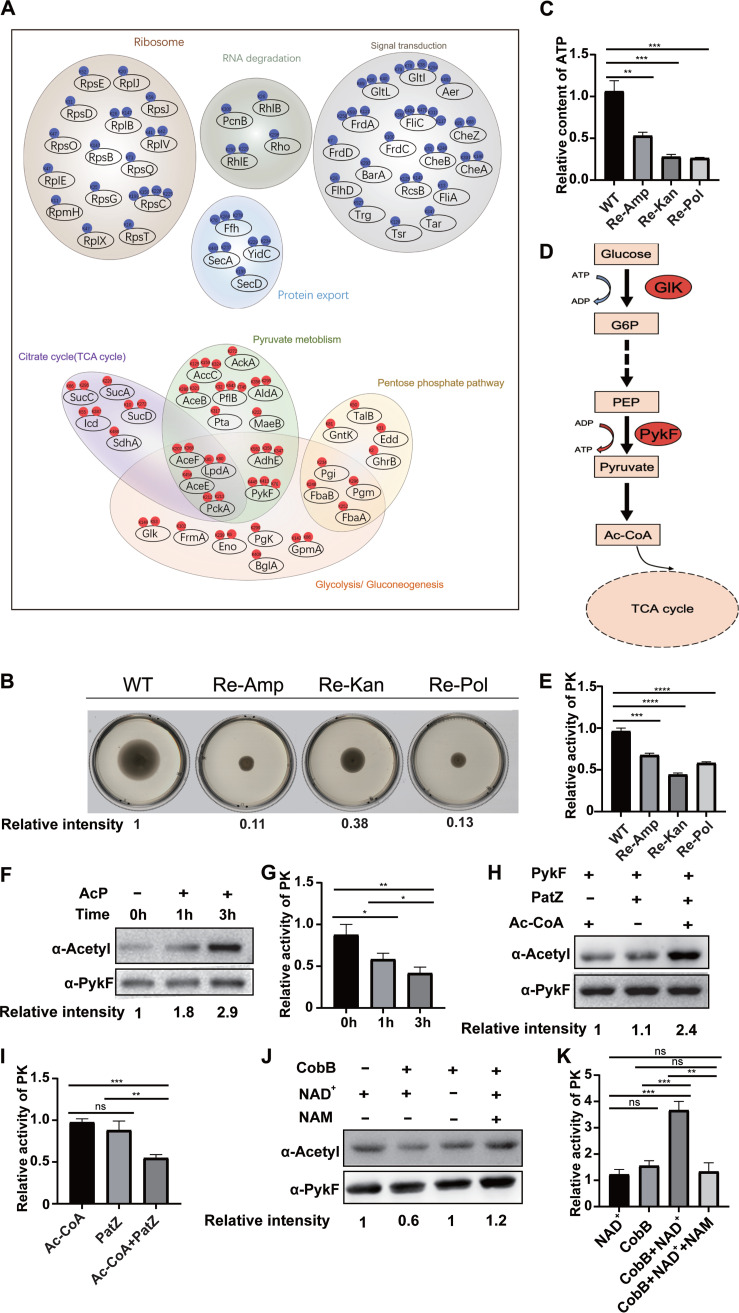
Acetylation negatively regulates the metabolism of antibiotic-resistant strains. (A) KEGG pathway analysis of the upregulated (Co-Up; red) and downregulated (Co-Down; blue) acetylated proteins in resistant bacteria. *P* < 0.05. (B) Determination of bacterial motility. Relative intensity of the bacterial movement area was measured by the ImageJ tool and calculated relative to the WT. (C) ATP content determination for resistant strains relative to WT. (D) Key kinases involved in the glycolysis process were overexpressed in the three resistant strains. Red oval represents upregulation in the resistant strains. (E) PykF enzyme activity detection relative to that of the WT. Experiments were performed in triplicate. (F) AcP acetylates PykF *in vitro*. Western blots are representative of at least three independent replicates. (G, I, K) PykF enzyme activity detection relative to that of the WT. (H) PatZ acetylates PykF *in vitro*. Western blots are representative of at least three independent replicates. (J) CobB deacetylates PykF *in vitro*. Two-tailed unpaired *t* test was used for statistical analysis. *, *P* < 0.05; **, *P* < 0.01; ***, *P* < 0.005; ****, *P* < 0.001.

To further investigate how lysine acetylation regulates bacterial resistance, we conducted a statistical analysis of the identified acetylation sites to locate common sites in the three resistant types. The acetylation levels of 185 acetylated proteins were upregulated (Co-Up), and those of 102 acetylated proteins were downregulated (Co-Down), in all three resistant strains compared with the WT. KEGG pathway analysis of these commonly changed acetylation sites showed that Co-Down acetylation mainly occurred in the ribosome, RNA degradation, signal transduction, and protein export (Fig. 3A). Lower levels of acetylation may inhibit signal transduction and prevent antibiotics from binding to the target proteins. Moreover, the acetylation levels of three flagellin proteins associated with bacterial movement were downregulated, including FliA, FlhD, and FliC (31, 32). Decreased bacterial motility is beneficial for bacteria to save energy and resist antibiotic pressure. In addition, we observed that Co-Up of acetylation sites mainly occurs in the TCA cycle, pyruvate metabolism, pentose phosphate pathway, and glycolysis/gluconeogenesis (Fig. 3A). To further validate the effects of protein acetylation on bacterial mobility and energy metabolism, we tested bacterial mobility and ATP levels. An agarose exercise analysis showed that the bacterial motilities of the three antibiotic-resistant strains were weaker than that of the WT (Fig. 3B). Meanwhile, the ATP content of the three antibiotic-resistant strains was significantly lower than that of the WT (Fig. 3C). Therefore, we suggest that diminished motility and reduced energy metabolism in resistant E. coli may be associated with acetylation of the related proteins.

Subsequently, we found that two key acetylated rate-limiting enzymes in glycolysis, Glk and PykF, were markedly upregulated in the three resistant strains and almost undetected in the WT (Fig. 3A and D). Among them, PykF, the key upstream kinase, catalyzes the reaction to produce ATP and pyruvate. Pyruvate is the substrate for forming acetyl-CoA, which affects the TCA cycle (33, 34). Therefore, PykF plays an important role in the process of energy metabolism. Thus, we tested the PykF enzyme activity of the WT and three resistant strains *in vitro* and found that PykF activity in the WT was significantly higher than that in the other antibiotic-resistant strains (Fig. 3E), indicating that PykF with high acetylation levels has a lower enzyme activity.

### PykF is reversibly acetylated by CobB and PatZ.

To unravel the role of PykF acetylation in bacterial resistance to antibiotics, we investigated the acetylation and deacetylation processes of PykF in E. coli, which has not been previously described. A previous study reported that protein acetylation in E. coli is mainly catalyzed by nonenzymatic AcP and peptidyl-lysine *N*-acetyltransferase (PatZ) (16). To study the acetylation mechanism of PykF, we expressed and purified the 6× His-tagged PykF protein and incubated it with AcP. An immunoblot with α-AcK antibody and the detection of PK enzyme activity indicated that nonenzymatic AcP could acetylate the purified PykF protein. Moreover, with an extended incubation time, the acetylation intensity of PykF increased, and its enzymatic activity decreased correspondingly (Fig. 3F and G). This result indicates that nonenzymatic AcP can acetylate PykF in E. coli, decreasing PykF enzyme activity.

Next, CobB and PatZ were also expressed, purified, and then incubated with PykF to determine their acetylation levels. The immunoblot results showed that PatZ could acetylate PykF in the presence of acetyl-CoA (Fig. 3H). Moreover, the increased acetylation level of PykF was accompanied by a decrease in enzymatic activity (Fig. 3I). In addition, when PykF was incubated with CobB and cofactor NAD^+^, PykF could be deacetylated by CobB. When the acetylation level of PykF decreased, its enzymatic activity increased accordingly (Fig. 3K). However, in the presence of nicotinamide (NAM; deacetylase inhibitor), PykF acetylation was not affected by CobB (Fig. 3J and K), indicating that PykF is a substrate of CobB. Understanding the acetylation and deacetylation processes of PykF is conducive to investigating the contribution of PykF acetylation to the regulation of energy metabolism and bacterial antibiotic resistance.

### K413 is the key acetylation site of PykF.

**FIG 4:**
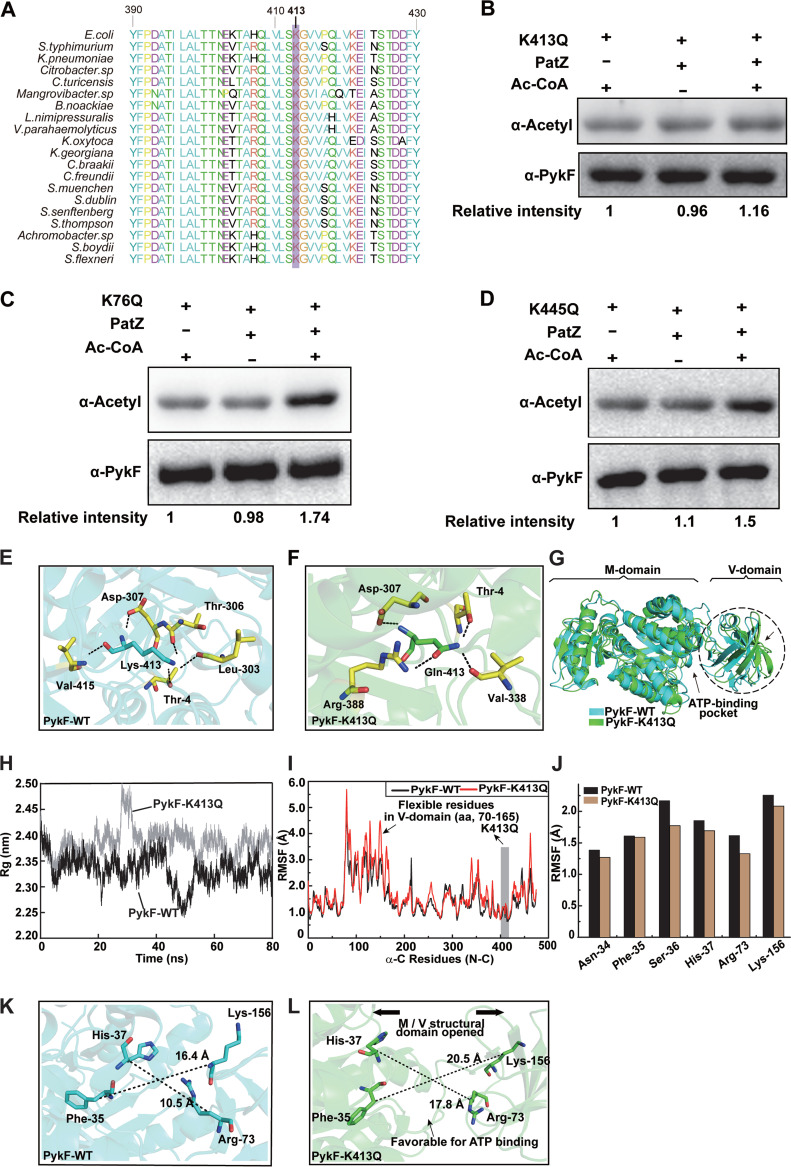
K413 of PykF is the acetylation site by PatZ. (A) Conservation analysis of PykF K413 in E. coli through sequence alignment. Purple frame denotes conserved lysine residues, and the result was analyzed by BioEdit v7.0. (B to D) PatZ acetylates K413Q, K76Q, and K445Q *in vitro*. (E) Hydrogen bonds between Lys-413 and the surrounding residues within 3.5 Å. (F) Hydrogen bonds between Gln-413 and its surrounding residues. (G) The overall display of protein structure includes two domains (M-domain and V-domain). Comparison of the average conformations of PykF-WT and PykF-K413Q in an equilibrium state. Dashed box represents the three-dimensional structure area with the largest conformational difference. The active center of ATP is marked by a black arrow. (H) Time evolution of the C-alpha of the radius of gyration (Rg) values. (I) The root-mean-square fluctuation (RMSF) values of the C-alpha for all residues of PykF-WT and PykF-K413Q proteins were calculated over the 80-ns trajectory. The RMSF value of residues in the V-domain of PykF-K413Q is higher than that of PykF-WT (black arrow). (J) RMSF of the C-alpha profiles of residues in the ATP active binding sites of PykF-WT and PykF-K413Q MD trajectories. (K to L) The differences in the ATP-binding pocket located between the V- and M-domains in PykF-WT or PykF-K413Q were compared, and the distances between the selected residues were measured.

To determine which lysine residues are acetylated in PykF that affect levels of the main acetylation changes, we analyzed the MS data, and three acetylation sites were found to be significantly upregulated in the three resistant strains, including K76, K413, and K445. The corresponding MS/MS spectra are shown in Fig. S1F. Among them, K413 was highly conserved in almost all the bacterial strains (Fig. 4A). To further test the roles of K76, K413, and K445 in PykF acetylation mediated by PatZ, these lysine residues were mutated to arginine (R) or glutamine (Q). The substitution of lysine for arginine avoids acetylation. However, it retains the positive charge, thus mimicking the nonacetylated form, while substituting glutamine mimics constitutive acetylation by neutralizing the positive charge (35). We hypothesized that if K413 is the key acetylation site of PykF, the acetylation level of this protein will not be affected by the acetyltransferase PatZ after the mutation of K413. When K413 was mutated to glutamine, we found that PykF could barely be acetylated by PatZ (Fig. 4B). However, when K76 and K445 were mutated to glutamine, respectively, PykF acetylation could still be enhanced by PatZ (Fig. 4C and D). These results indicate that K413 is a key site of acetylation by PatZ in PykF, while K76 and K445 are not. Thus, we speculated that K413 contributes to PykF activity by affecting the acetylation level.

To further investigate the effects of Lys-413 acetylation on PykF conformation and its enzymatic activity, we analyzed the conformational differences of PykF-WT and PykF-K413Q in an equilibrium state using molecular dynamics (MD) simulation. The results showed that five hydrogen bonds formed between Lys-413 and the surrounding residues Thr-4/Leu-303/Thr-306/Asp307/Val-415 in PykF (Fig. 4E). At the same time, Gln-413 interacted with Thr-4/Asp307/Val-338/Arg-388 in PykF-K413Q (Fig. 4F), indicating that the replacement of Lys-413 by Gln induced the local microenvironment change in the key acetylated site.

By observing the whole conformation of PykF-WT, we found that about 96 residues (amino acids [aa], 70 to 165) formed a single Vice-domain (V-domain), while the remaining residues formed the main-domain (M-domain) (Fig. 4G). These two domains jointly contribute to the biological activity of pyruvate kinase. According to the description of this protein in the UniProt database, the ATP pocket is located at the interface between the M- and V-domains. Thus, we merged the conformations of PykF-WT and PykF-K413Q under an equilibrium state and found that the overall conformation of the V-domain deviated from that of the M-domain in the mutant protein, which was conducive to the ATP pocket showing an opened state, facilitating ATP binding. However, ATP inhibits PykF activity because PykF in E. coli is a type I isozyme (36–38).

Based on the results of MD simulation, we found that the radius of gyration (Rg) values of C-alpha of PykF-K413Q were larger than those of PykF-WT (Fig. 4H), indicating that replacing Lys-413 by simulating Gln may induce conformational looseness in the whole protein. Moreover, the residues of the V-domain of PykF-K413Q showed higher root-mean-square fluctuation (RMSF) values than those of PykF-WT (Fig. 4I), indicating that the residues of the V-domain are more flexible in the mutant protein. We further calculated the RMSF values of the typical ATP-binding site residues Asn-34/Phe-35/Ser-36/His-37/Arg-73/Lys-156 of PykF and found that they were more stable in PykF-K413Q than in PykF-WT (Fig. 4J). In addition, the induced conformation dramatically increased distances between the ATP-binding site residues in PykF, specifically between Phe-35 and Lys-156 and between His-37 and Arg-73 (Fig. 4K and L), resulting in a larger solvent-accessible surface area of the ATP active center and longer distances between the M- and V-domains which jointly contribute to ATP binding. These results suggested that all these conformational changes induced by acetylation may promote the binding of PykF-K413Q with ATP, further inhibiting the enzyme activity.

### Mimic deacetylation of PykF K413 reduces bacterial resistance ability.

PykF is an important key rate-limiting enzyme for ATP production during glycolysis which provides pyruvate for the TCA cycle (33). Interestingly, we identified K413 acetylation of PykF in all three resistant strains but not in the WT. Thus, we speculated that K413 acetylation could contribute to regulating resistance in bacteria. To study whether the deacetylation of the K413 site affects drug resistance, we knocked out the *pykF* gene in the three resistant strains to construct Δ*pykF*-Amp/Kan/Pol mutant strains. We then introduced the plasmid with wild-type PykF, PykF-K413Q, and PykF-K413R genes into cells to obtain eWT-Amp/Kan/Pol, eK413Q-Amp/Kan/Pol, and eK413R-Amp/Kan/Pol complementary strains, respectively. At the same time, to make the experiment more clinically meaningful, we used the same method to knock out the *pykF* gene in a clinical multidrug-resistant (CMR) E. coli strain (clinical strain no. 966113) and then introduced the three plasmids described above into it, resulting in eWT-CMR, eK413Q-CMR, and eK413R-CMR.

**FIG 5:**
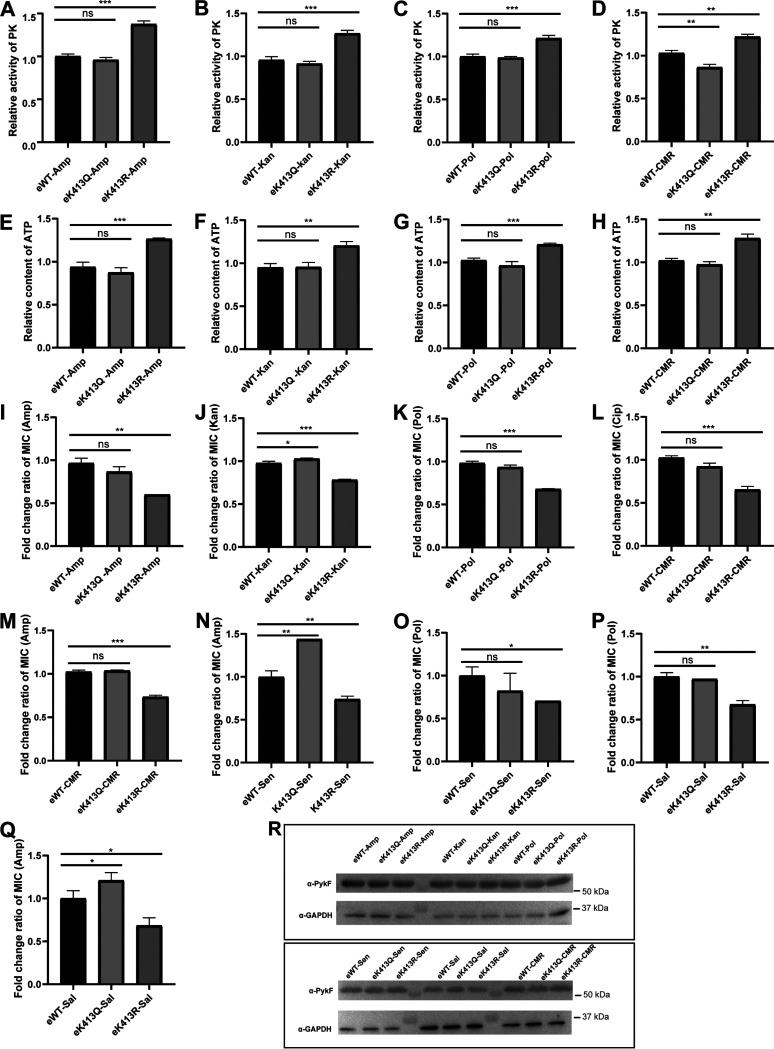
Deacetylation of PykF K413 reduces E. coli resistance. (A to D) PykF enzyme activity determination. (E to H) ATP content determination. (I to M) MIC determination of the resistant strains in which *pykF* was knocked out and then complemented with wild-type *pykF*, K413Q, and K413R plasmid. (N to O) MIC change in sensitive E. coli BW25113 with *pykF* knocked out. (P to Q) MIC changes of Salmonella with wild-type or mutant PykF to ampicillin and polymyxin. (R) PykF expression levels in the different complementary strain were determined by Western blotting. Experiments were performed in triplicate. Two-tailed unpaired *t* test was used for statistical analysis. *, *P* < 0.05; **, *P* < 0.01; ***, *P* < 0.005; ****, *P* < 0.001.

We collected the constructed bacteria at OD_600_ = 0.8 and determined their PykF enzyme activity and ATP content in cells. The results showed that in the four resistant strains, K413 mimic deacetylation resulted in increased PykF enzyme activity (Fig. 5A to D) and ATP content (Fig. 5E to H). Western blot (WB) detection showed that the PykF expression levels in the three complementary strains of each resistant strain were similar (Fig. 5R). Importantly, it was found that the supplement with PykF-K413R decreased the antibiotic resistance of the three resistant strains obtained in the laboratory (Fig. 5I to K) and the CMR strains to ampicillin and ciprofloxacin (Fig. 5L and M). In addition, we also carried out the corresponding experiments in the sensitive strain E. coli BW25113 (Fig. 5N and O) and obtained the same conclusion as above. On the other hand, we directly expressed WT PykF, PykF K413R, and PykF K413Q in sensitive Salmonella ATCC14028 and determined their MICs. We found that PykF-K413R was also associated with resistance of Salmonella to ampicillin and polymyxin (Fig. 5P and Q). These results indicate that K413 is an important lysine acetylation site whose deacetylation increases PykF activity and ATP production, correlating with decreased bacterial resistance to antibiotics.

**FIG 6:**
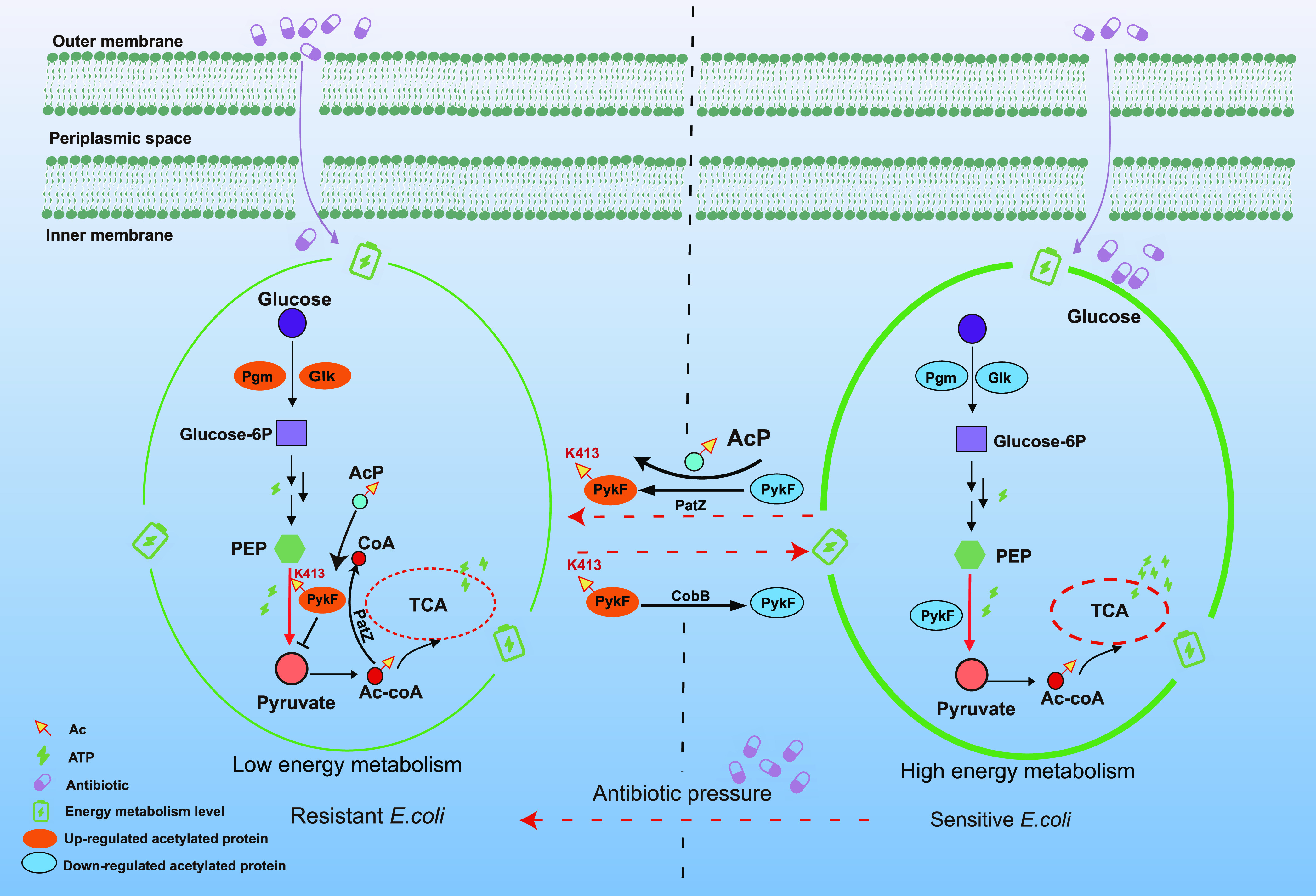
Illustration of lysine acetylation regulating bacterial resistance. Under long-term pressure from antibiotics, E. coli acquired drug resistance through acetylation negative regulation of its own metabolism. In drug-resistant bacteria, it was found that the acetylation of lysine at position 413 of pyruvate kinase PykF in the pyruvate cycle decreased its enzyme activity and inhibited the TCA cycle to maintain a low-energy metabolism. In this study, it was found that deacetylation of PykF 413K could restore the sensitivity of E. coli to antibiotics (kanamycin, ampicillin, polymyxin B) and increase its energy metabolism.

In summary, with immune enrichment and DIA-based quantitative proteomics, we found a common regulatory mechanism of antibiotic resistance by acetylation in bacteria. Lysine acetylation negatively regulates energy metabolism and positively regulates bacterial motility, reducing the overall metabolic level of bacteria to modulate drug resistance. PykF, the key limiting-rate kinase in glycolysis, was highly acetylated in the three resistant strains, and K413 was found to be an important acetylation site for this enzyme activity. Changes in the acetylation state to reduce levels of PykF K413 acetylation increases bacterial energy metabolism and the sensitivity of bacteria to antibiotics. Therefore, this study uncovers a new systematic regulatory mechanism for drug-resistant strains (Fig. 6) and provides novel insights for the development of new antibacterial drugs.

## DISCUSSION

Over the past decades, the abuse of antimicrobial drugs has led to the appearance of various super-resistant and multi-resistant strains which have seriously endangered human health (39, 40). Therefore, in recent years, the prevention and treatment of infection caused by drug-resistant strains and the rational use of antibiotics have received worldwide attention. A systematic study of the mechanisms of bacterial resistance is necessary to combat the infections caused by drug-resistant strains. The development of bacterial resistance is a process in which multiple mechanisms work together (1, 39–41). Under the pressure of antibiotics, bacteria can gradually adapt to the environment by regulating their physiological state, thus obtaining drug resistance (42, 43). Meanwhile, more and more studies have pointed out that changes in bacterial metabolic levels can significantly affect the bactericidal effect of antibiotics (44, 45). Post-translational modifications of proteins are involved in many important metabolic pathways, including the regulation of key catalytic enzymes.

We conducted quantitative acetylation proteomics research to explore the relationship between acetylation and bacterial resistance. It is notable that certain specific acetylated proteins were only detected in one type of resistant strain, suggesting that different antibiotic-resistant strains have specific acetylated proteins involved in the regulation of resistance. Statistical analysis of these unique acetylated proteins showed that 13 specific acetylated proteins were involved in the resistance regulation of ampicillin-resistant strains, 28 were observed in kanamycin-resistant strains, and 58 were involved in polymyxin B-resistant strains. According to different biological processes, specific acetylated proteins which may be related to different antibiotic resistances were determined (Table S3). For example, acetylated entericidin B (EcnB), a membrane-associated protein, was only identified in ampicillin-resistant strains. EcnB plays a role in bacteriolysis and affects the synthesis of membrane proteins. Changes in membrane protein structure might affect the efficacy of ampicillin (2), indicating that EcnB acetylation may be uniquely involved in regulating ampicillin resistance. In addition, the unique acetylated protein AtoC, a member of the two-component regulatory system AtoS/AtoC, was only found in kanamycin-resistant strains. AtoC acts not only as a transcriptional regulator but also as a post-translational regulator (46, 47). It can inhibit the biosynthesis of polyamines by regulating ornithine decarboxylase. However, because the target site of kanamycin is the 30S subunit, we speculated that AtoC acetylation might inhibit polyamine biosynthesis, inhibiting the complete structure formation of the 30S subunit. This change in the target site might lead to bacterial resistance to kanamycin. Additionally, in polymyxin B-resistant strains, we found that the unique acetylated proteins ArnA and ArnB modify lipid A to change the target site of polymyxin B attack (48, 49). Therefore, these two unique acetylated proteins may be involved in regulating bacterial resistance to polymyxin B. These highly acetylated proteins indicate that different resistant strains may have distinct regulatory mechanisms. Moreover, these results indicate that these proteins may be used as biomarkers to identify different types of resistant strains; however, further research and verification are still needed.

10.1128/msystems.00649-22.3 TABLE S3 Primers used in this study. Download Table S3, DOCX file, 0.01 MB. Copyright © 2022 Fang et al. 2022 Fang et al. https://creativecommons.org/licenses/by/4.0/ This content is distributed under the terms of the Creative Commons Attribution 4.0 International license.

More importantly, bioinformatics analysis showed a common characteristic in the three resistant strains: acetylation can regulate E. coli metabolism in two ways to maintain antibiotic resistance. First, acetylation positively regulates bacterial motility by reducing the acetylation level of the flagellar movement-related proteins, of which FliA and FlhD were identified among the Co-Down-regulated DAPs. FlhD is an upstream transcription factor that can form the transcription activation complex FlhD4C2 with FlhC. FlhD integrates many input signals from global regulatory factors and determines cellular mobility (32, 50). In addition, FlhD can regulate the expression of FliA, which significantly affects biofilm formation ability (51), indicating that mobility is also an important factor affecting biofilm formation. Moreover, the biofilm contributes to bacterial resistance. Therefore, we believe that a decrease in the acetylation levels of FlhD and FliA proteins may promote the development of bacterial resistance. However, the mechanism behind specific control needs further verification. Second, acetylation negatively regulates the energy metabolism of bacteria by increasing the acetylation levels of central metabolism-related proteins. Previous studies have shown that regulation of bacterial metabolic levels can improve the resistance efficacy of antibiotics. For example, the addition of exogenous glutamic acid can increase the sensitivity of bacteria to aminoglycoside antibiotics (44), but the related metabolic regulation mechanism is still unclear. Therefore, from the point of view of lysine acetylation, we compared the WT with the resistant strains to explore the similarities in resistance mechanisms. The Co-Up of DAPs was significantly enriched in the central metabolic regulation process, which is consistent with the previously reported acetylation concentrated in the carbon metabolism regulation process (12, 16, 24).

In addition, the pyruvate cycle (oxaloacetate-PEP-pyruvate-acetyl-CoA) can produce a large amount of energy. When the enzymes in the pyruvate cycle are inhibited, or genes are inactivated, the TCA cycle is blocked (44). PykF is the key kinase in the pyruvate cycle, which is essential for the production of ATP and pyruvate and affects levels of energy metabolism in bacteria (44). Our study found that the acetylated form of PykF was significantly upregulated in the three drug-resistant strains. Moreover, our additional studies found that its enzyme activity is negatively regulated by acetylation, and the K413 site is a highly conserved key acetylation site. Deacetylation at this site can increase the enzymatic activity of PykF, increasing energy metabolism in bacteria, which in turn increases the sensitivity of drug-resistant strains to antibiotics. The same was observed even in the clinical multi-resistant strains to a certain level. However, compared with that of sensitive bacteria, sensitivity to antibiotics cannot be fully restored; that is, the strain still retains a certain degree of resistance due to the other energy compensation mechanisms in bacteria.

In conclusion, we found that lysine acetylation exhibits similarities in antibiotic-resistant strains with different resistances. By positively regulating bacterial motility and negatively regulating energy metabolism, it reduces the metabolic level of bacteria to regulate antibiotic resistance. Furthermore, our research found that deacetylation of the K413 site of the PykF protein can increase the enzyme activity of PykF, leading to increased energy metabolism in bacteria, which in turn increases the sensitivity of antibiotic-resistant strains to antibiotics. The metabolic network of lysine acetylation to regulate bacterial resistance has been described here, and key regulatory proteins were detected which provide a new perspective for studying bacterial resistance mechanisms.

## MATERIALS AND METHODS

### Detection of antibiotic resistance of bacterial strains.

All bacterial strains, plasmids, and primers used in this study are listed in Tables S1 and S2. Amp’s MIC was determined via microdilution in a 48-well plate. The bacteria were diluted to an OD_600_ of 0.05 in Luria-Bertani (LB) broth. Then, 500 μL of diluted culture was added to each well of a 48-well plate containing ampicillin (Amp) at different concentrations. Cultures were incubated at 37°C for 24 h, and growth was detected using a microplate reader. The drug concentration in which the bacterial growth of an OD_600_ below 0.1 was observed was recorded as the MIC (52). Three single colonies of WT E. coli BW25113 were cultured overnight and then diluted 1:100 in fresh LB medium with or without 0.5× MIC of Amp. After 24 h of incubation at 37°C with agitation, the MIC was determined again. Next, the bacteria were continuously subcultured at sub-MIC and the concentration of antibiotics was gradually increased to improve the resistance of the bacteria. The same procedure was repeated until the MIC of Amp increased to 481.7 μg/mL; 60-fold greater than the original MIC. The kanamycin- and polymyxin B-resistant E. coli strains were obtained using the same method. The development of resistance ability of the Δ*patZ* and Δ*cobB* strains to ampicillin and polymyxin B was also tested using the method described above.

10.1128/msystems.00649-22.1 TABLE S1 Specific acetyl proteins identified in this study. Download Table S1, DOCX file, 0.01 MB. Copyright © 2022 Fang et al. 2022 Fang et al. https://creativecommons.org/licenses/by/4.0/ This content is distributed under the terms of the Creative Commons Attribution 4.0 International license.

### Cell culture and protein exaction.

All strains used here were activated in LB medium at 37°C overnight, transferred into fresh LB medium (1:100, vol/vol) for culturing, and harvested after the OD_600_ approached 0.8 through centrifugation at 8,000 × *g* for 10 min at 4°C. The cell pellets were washed three times with ice-cold phosphate-buffered saline (PBS) and then sonicated in 500 μL of SDS lysis buffer supplemented with protease inhibitor cocktail (Roche, Basel, Switzerland), 1 mM phenylmethanesulfonyl fluoride (PMSF), and deacetylase inhibitor mix (Beyotime, Nanjing, China). After centrifugation at 12,000 × *g* for 30 min, the supernatant was collected. Trichloroacetic acid at a final concentration of 20% was added to the supernatant, which was left at 4°C for 2 h. The solution was centrifuged at 4°C and 12,000 × *g* for 20 min, and the precipitation was obtained. The precipitation was rinsed 3 times with acetone pre-cooled at −20°C. Finally, acetone was removed by solution centrifugation, and precipitation was re-dissolved with 8 M urea. The protein concentration was determined using the BCA Protein Assay kit (Thermo Fisher Scientific, Shanghai, China).

### Western blotting and competition experiment.

The protein from each sample was loaded into the 12% SDS-PAGE gel and then transferred to a polyvinylidene fluoride (PVDF) membrane (MilliporeSigma, USA). Acetylated antibody (PTM BioLab, PTM-101) was incubated with a PVDF membrane at 4°C, and horseradish peroxidase-conjugated goat anti-mouse was used as a secondary antibody. The results were visualized with Clarity Western ECL Substrate (Bio-Rad, USA) and captured with ImageMaster2D Platinum 6.0 (GE Healthcare, USA). Meanwhile, SDS-PAGE gel stained with Coomassie brilliant blue R250 was used as the loading control.

The competition experiment was performed in accordance with a previously described method (53). The peptide library bearing unmodified lysine or the peptide library bearing acetyllysine (KAc) was synthesized at ChinaPeptides Co., Ltd.

### Protein digestion and affinity enrichment of acetylated peptides.

Protein digestion and affinity enrichment of the acetylated peptides were performed as described in a study conducted by us previously (27). In brief, the protein extract was reduced with 8 M urea and 50 mM dithiothreitol (DTT; 37°C, 1 h) and alkylated with 100 mM iodoacetamide (IAA; 25°C, 30 min). Samples were transferred into the 30-kDa ultracentrifugal filters (Sartorius Stedim Biotech, Shanghai, China) and washed thrice with 8 M urea and 50 mM triethylammonium bicarbonate buffer (TEAB). Protein and trypsin were mixed at a mass ratio of 30:1 for digestion at 37°C for 16 h. The peptides were then lyophilized at −80°C for further analysis. An α-AcK antibody beaded agarose kit (PTM Biolabs, Hangzhou, China) was used to enrich acetylated peptides. The lyophilized peptide was dissolved in 200 μL of pre-cold immunoprecipitation buffer, then gently mixed with antibody-conjugated beads. The ratio of peptides to beads was 3 mg peptides to 30 μL drained antibody beads. After incubating at 4°C overnight, the peptides were washed with wash buffer I and wash buffer II thrice, and the acetylated peptides were eluted with 100 μL of elution buffer. Finally, the elutes were dried for MS analysis.

### Bioinformatics analysis.

To obtain the actual change intensity of acetylation, we used the following formula to determine the protein background: (A/a)/(B/b), where ‘A’ represents the quantitative acetylation value of resistant strains, ‘a’ represents the quantitative protein value of resistant strains, ‘B’ represents the WT strain quantitative acetylation value, and ‘b’ represents the WT strain quantitative protein value. In this way, we obtained the up-down fold-change relative to the WT strain and the subsequent data analysis.

Gene Ontology (GO) enrichment analysis was performed in Blast2GO (54) with Fisher’s exact test of false discovery rate (FDR) of <0.05. Kyoto Encyclopedia of Genes and Genomes (KEGG) pathway enrichment analysis was performed on the “Wu Kong” platform. Functional protein domains were predicted with the Pfam 31.0 database (55). The “Wu Kong” platform was also used to analyze amino acid sequences and generate sequence logos.

### Cloning, expression, and purification of target proteins.

*PykF*, *cobB*, and *patZ* genes were amplified from the genomic DNA of E. coli BW25113. The PCR product and pET28b plasmid were digested with restriction endonuclease EcoRI and HindIII (TaKaRa Bio, Japan), then ligated with T4 DNA Ligase (TaKaRa Bio) and transformed into E. coli BL21 The strains verified by sequencing were cultured in LB medium containing 50 μg/mL kanamycin at 37°C. When OD_600_ reached 0.6, 0.5 mM isopropyl-d-thiogalactoside (IPTG; Sigma-Aldrich, USA) was added and cultured for 5 h to induce protein expression. The bacteria were centrifuged for 8,000 × *g* at 4°C and washed 3 times with 0.01 M PBS. The collected cells were crushed with a 0.01 M PBS suspension and cracked under high pressure. The supernatant was obtained by centrifugation for 30 min at 12,000 × *g*. Finally, the recombinant protein was purified by an Ni-NTA affinity chromatography column (Qiagen, Germany).

### Construction of gene deletion and complementary strain.

Knockout of *pykF* was accomplished by methods resembling those used in the Keio knockout collection (56, 57). The primers used to clone homologous *pykF* for construction of these mutants are listed in Table S2. The pCas9 plasmid was electrotransferred into prepared competent cells, and the monoclonal cells were picked out and cultured in LB medium at 30°C. The monoclonal cells were then induced with arabinose for Cas9 protein expression and prepared as competent cells into which the homologous fragments and psgRNA plasmids were transferred, and then coated on gentamicin-resistant culture plates and cultured in an incubator at 30°C. The gene deletion mutants were confirmed by PCR. The deletion mutant was confirmed by PCR. The test primers are listed in Table S2.

10.1128/msystems.00649-22.2 TABLE S2 Strains and plasmids used in this study. Download Table S2, DOCX file, 0.01 MB. Copyright © 2022 Fang et al. 2022 Fang et al. https://creativecommons.org/licenses/by/4.0/ This content is distributed under the terms of the Creative Commons Attribution 4.0 International license.

To construct the complementary strains, the constructed pET28b-pykF, pET28b-pykF (K413Q), and pET28b-pykF (K413R) plasmids were amplified by PBSU101 primers (Table S2), and the PBSU101 plasmid was digested by BamHI and XbaI (TaKaRa Bio, Japan). Next, PBSU101-pykF, PBSU101-pykF (K413Q), and PBSU101-pykF (K413R) were obtained by homologous recombination (ClonExpress II One-Step Cloning kit, Vazyme Biotech, China) of the amplified product and the digested linearized plasmid. Next, the obtained plasmids were introduced into the corresponding strains.

### Site-directed mutagenesis of pykF.

A Mut Express II Fast Mutagenesis kit V2 (Vazyme, cat. no. C214-01) was used to introduce base substitutions into the WT *pykF* allele through the corresponding primers according to the manufacturer’s instructions. All site-directed mutants were confirmed by DNA sequencing.

### *In vitro* modification assay.

All *in vitro* modification assays were performed as described in previous studies (21, 58). For the PatZ modification assay, PykF (0.2 μg/μL) was purified and incubated at 37°C for 6 h in the presence or absence of PatZ (0.2 μg/μL) and acetyl-CoA (0.2 mM). For the CobB deacetylation assay, PykF (0.4 μg/μL) protein was incubated at 30°C for 8 h in the presence or absence of CobB (0.2 μg/μL), NAD^+^ (3 mM), and NAM (30 mM). For the AcP modification assay, PykF was incubated at 37°C in the presence or absence of 20 mM AcP, and the samples were collected after 0 and 3 h.

### Motility assays.

The motility assays were performed as described in our previous study (59). Bacterial solution (1 μL) was inoculated at the center of an agarose plate (Swimming plate) and incubated at 37°C for 24 h. Next, the diffusion areas of different bacteria on the plate were calculated using Image J software (National Institutes of Health, Bethesda, MD, USA).

### ATP and pyruvate kinase activity assays.

ATP levels and pyruvate kinase activity were determined using the ATP assay kit (Beyotime, China cat. no. S0026) and Pyruvate kinase assay kit (Solarbio Life Science, China, cat. no. BC0540), respectively, according to the manufacturer’s instructions.

### MD simulation.

In this study, the MD simulations were carried out in the 80-ns trajectory using the Gromacs 2019.5 package with the SPC water model in the Gromos43a1 force field. PyMOL software was used to perform the mutation from Lys-413 to Gln-413. Next, the PDB structures of PykF-WT and PykF-K413Q were used as the starting conformations in one cubic periodic box, and the minimum distance between the protein and the box boundary was set at 1.0 nm. The periodic boundary condition of the MD system was suitable for the three directions of X, Y, and Z. The solute (0.15 mol/L NaCl salt) was added in solvent to neutralize the system. Electrostatic interactions were computed using the particle mesh ewald (PME) method, and the atomic motion was calculated using the leapfrog algorithm within the MD system. The 400-step energy minimization was performed using the steepest descent energy method. Next, we carried out the 25,000-step energy minimization using the conjugate gradient method and the 50-ps position restraint simulation in each system. Each MD trajectory was carried out with a random initial velocity at a constant temperature of 300 K.

### MD trajectory analysis.

The root mean square fluctuations of each alpha-C of the residue was calculated using the gmx rmsf tool, and the Rg values of atomic evolution with the simulation time were calculated using the gmx gyrate tool of Gromacs v2019.5. The conformational changes between PykF-WT and PykF-K413Q were observed from the MD simulations trajectory by using VMD-1.9.1 software. Next, the average distances between the typical ATP binding site residues of PykF-WT and PykF-K413Q, such as those between Phe-35 and Lys-156 and between His-37 and Arg-73, respectively, were measured by gmx distance. The hydrogen bonds around Lys-413 or Gln-413 were analyzed using gmx hbond, and PyMOL and Origin 8.5 software were used to draw the structure diagram.

### Data availability.

The mass spectrometry proteomics data have been deposited to the ProteomeXchange Consortium via the PRIDE (60) partner repository with the data set identifier PXD030283.
