# Supplementary material for: Potential Role of Lysine Acetylation in Antibiotic Resistance of Escherichia coli
Source: mSystems. 2022 Oct 26;7(6):e00649-22. doi: 10.1128/msystems.00649-22 (PMC9765299; doi:10.1128/msystems.00649-22)
Supplement: TABLE S3 [file msystems.00649-22-s0004.docx]

**Table S3. Specific acetyl proteins identified in different resistance strains**

| **Specific acetylated protein related to ampicillin resistance** | |
| --- | --- |
| **Characteristics** | **Acetylated proteins and sites** |
| Multidrug resistance protein | MdlA K232 |
| Membrane lipoprotein | EcnB K45 |
| DNA binding protein | DecR K115, YrdD K123 |
| Stimulus sensor | InaA K176 |
| Glucose dehydrogenase | Gcd K274/K685 |
| Sugar phosphotransferase system | Yadl K82 |
| **Specific acetylated protein related to kanamycin resistance** | |
| **Characteristics** | **Acetylated proteins and sites** |
| Two-component system | AtoC K126/K127, NarL K100 |
| ABC transporter | HisJ K120 |
| Kinase | NanK K197 |
| Membrant protein | YejM K220, TolA K10, MinC K26 |
| Toxin-antitoxin system | DinJ K73 |
| **Specific acetylated protein related to polymyxin resistance** | |
| **Characteristics** | **Acetylated proteins and sites** |
| Polymyxin resistance protein | ArnA K181/K422/K442/K458/K532/K611,  ArnB K213/K337 |
| LPS related enzymes | Ais K76/K188, EptC K9/K547 |
| Toxin-antitoxin system | YhaV K73 |
| Kinase | Tmk K4/K209, PdxY K108/K120 |
| Two-component system | BasR K164, UvrY K41, YehU K327 |
| ABC transporter | HisP K148 |
| DNA binding protein | Ssb K88, MutY K103, SlmA K179, StpA K107 |
| Reductase | Fre K83, ProA K15, ProC K226, YbjS K334 |
| Iron metabolism related protein | EntC K237, Fes K47, IscU K46, IscA K89, MenF K120 |
