# Supplementary material for: Potential Role of Lysine Acetylation in Antibiotic Resistance of Escherichia coli
Source: mSystems. 2022 Oct 26;7(6):e00649-22. doi: 10.1128/msystems.00649-22 (PMC9765299; doi:10.1128/msystems.00649-22)
Supplement: TABLE S1 [file msystems.00649-22-s0002.docx]

**Table S1. Strains and plasmids used in this study.**

| **Strains and plasmids** | **characteristics** | **Sources or references** |
| --- | --- | --- |
| **Strains** | | |
| *E. coli BL21* | Expression strain | Laboratory stock |
| *E. coli BW25113* | Wild type *Escherichia coli* strain | Laboratory stock |
| *Salmonella ATCC14028* | Wild type *Salmonella* strain | Laboratory stock |
| Re-Amp | *Ampicillin resistant Escherichia coli strain* | This study |
| Re-Kan | *Kanamycin resistant Escherichia coli strain* | This study |
| Re-Pol | *Polymyxin B resistant Escherichia coli strain* | This study |
| Δ*pykF* | Wild type Escherichia coli BW25113 strain Δ*pykF* | NBRP (NIG, Japan) |
| Δ*cobB* | Wild type Escherichia coli BW25113 strain Δ*cobB* | NBRP (NIG, Japan) |
| Δ*patZ* | Wild type Escherichia coli BW25113 strain Δ*patZ* | NBRP (NIG, Japan) |
| Δ*pykF:: Re-Amp* | Ampicillin resistant Escherichia coli strain ΔpykF | *This study* |
| Δ*pykF:: Re-Kan* | *Kanamycin resistant Escherichia coli strain ΔpykF* | This study |
| Δ*pykF:: Re-Pol* | *Polymyxin B resistant Escherichia coli strain ΔpykF* | This study |
| Δ*pykF:: CMR* | *Multidrug resistant Escherichia coli strain ΔpykF* | This study |
| eWT-Amp | *PBSU101 with PykF, overexpressed in ΔpykF:: Re-Amp* | This study |
| eWT-Kan | *PBSU101 with PykF, overexpressed in ΔpykF:: Re-Kan* | This study |
| eWT-Pol | *PBSU101 with PykF, overexpressed in ΔpykF:: Re-Pol* | This study |
| eWT-Sen | *PBSU101 with PykF, overexpressed in ΔpykF* | This study |
| eWT-Sal | *PBSU101 with PykF, overexpressed in Salmonella* | This study |
| eWT-CMR | *PBSU101 with PykF, overexpressed in ΔpykF:: CMR* | This study |
| eK413Q -Amp | *PBSU101 with PykF (K413Q), overexpressed in ΔpykF:: Re-Amp* | This study |
| eK413Q-Kan | *PBSU101 with PykF (K413Q), overexpressed in ΔpykF:: Re-Kan* | This study |
| eK413Q-Pol | *PBSU101 with PykF (K413Q), overexpressed in ΔpykF:: Re-Pol* | This study |
| eK413Q-Sen | *PBSU101 with PykF (K413Q), overexpressed in ΔpykF* | This study |
| eK413Q-Sal | *PBSU101 with PykF (K413Q), overexpressed in Salmonella* | This study |
| eK413Q-CMR | *PBSU101 with PykF (K413Q), overexpressed in ΔpykF:: CMR* | This study |
| eK413R-Amp | *PBSU101 with PykF (K413R), overexpressed in ΔpykF:: Re-Amp* | This study |
| eK413R-Kan | *PBSU101 with PykF (K413R), overexpressed in ΔpykF:: Re-Kan* | This study |
| eK413R-Pol | *PBSU101 with PykF (K413R), overexpressed in ΔpykF:: Re-Pol* | This study |
| eK413R-Sen | *PBSU101 with PykF (K413R), overexpressed in ΔpykF* | This study |
| eK413R-Sal | *PBSU101 with PykF (K413R), overexpressed in Salmonella* | This study |
| eK413R-CMR | *PBSU101 with PykF (K413R), overexpressed in ΔpykF:: CMR* | This study |
| **Plasmids** | | |
| pET28b | Expression vector | Laboratory stock |
| pET28b-*pykF* | *pykF in pET28b* | This study |
| pET28b-*pykF* (K413Q) | *pykF (K413Q) in pET28b* | This study |
| pET28b-*pykF* (K413R) | *pykF (K413R) in pET28b* | This study |
| PBSU101-*pykF* | *pykF in PBSU101* | This study |
| PBSU101-*pykF* (K413Q) | *pykF (K413Q) in PBSU101* | This study |
| PBSU101-*pykF* (K413R) | *pykF (K413R) in PBSU101* | This study |
| pET28b-*cobB* | *cobB in pET28b* | This study |
| pET28b-*patZ* | *patZ in pET28b* | This study |
