# Supplementary material for: Potential Role of Lysine Acetylation in Antibiotic Resistance of Escherichia coli
Source: mSystems. 2022 Oct 26;7(6):e00649-22. doi: 10.1128/msystems.00649-22 (PMC9765299; doi:10.1128/msystems.00649-22)
Supplement: TABLE S2 [file msystems.00649-22-s0003.docx]

**Table S2. Primers and peptides used in this study.**

| **Primer** | **Sequence** |
| --- | --- |
| pET28b-pykF-EcoRI-F | *ccggaattcgatgaaaaagaccaaaattgt* |
| pET28b-pykF-HindIII-R | *cccaagcttttacaggacgtgaacagatgc* |
| pET28b-cobB-EcoRI-F | *ccggaattcgatgctgtcgcgtcggggtcatcg* |
| pET28b-cobB-HindIII-R | *cccaagctttcaggcaatgcttcccgctt* |
| pET28b-patZ-EcoRI-F | *ccggaattcgatgagtcagcgaggactggaagc* |
| pET28b-patZ-HindIII-R | *cccaagctttcatgattcctcgcgctgggcaag* |
| *pykF* K413Q-F | *tactgagccaaggcgttgtgccgcagc* |
| *pykF* K413Q-R | *aacgccttggctcagtaccaactgatgagccg* |
| *pykF* K413R-F | *tactgagcagaggcgttgtgccgcagct* |
| *pykF* K413R-R | *aacgcctctgctcagtaccaactgatgagcc* |
| △PykF-up-F | *tttattggctaatgctgtacgtaa* |
| △PykF-up-R | *ttcacaaaagcaatagacagtcttagtctttaagtt* |
| △PykF-down-F | *gactaagactgtctattgcttttgtgaattaatttg* |
| △PykF-down-R | *gcagccagcaatgcgccttcag* |
| △PykF-sgRNA | *gctatcctgcttgataccaa* |
| △PykF-JD-F | *tcagcgtataatgcgcgccaattga* |
| △PykF-JD-R | *ataccctctagattgagttaatctc* |
| △PykF-ter-F | *atgaaaaagaccaaaattgtttgca* |
| △PykF-ter-R | *ttacaggacgtgaacagatgcggtg* |
| PBSU101-F | *gttaaacatatgatggatccatgaaaaagaccaaaattgtttgc* |
| PBSU101-R | *tctagattacaggacgtgaacagatgcg* |
